# Supplementary material for: Ocular findings, surgery details and outcomes in proliferative diabetic retinopathy patients with chronic kidney disease
Source: PLoS One. 2022 Oct 21;17(10):e0273133. doi: 10.1371/journal.pone.0273133 (PMC9586353; doi:10.1371/journal.pone.0273133)
Supplement: S1 Checklist — (DOCX) [file pone.0273133.s001.docx]

STROBE Statement—checklist of items that should be included in reports of observational studies

|  | Item No. | Recommendation | Page  No. | Relevant text from manuscript |
| --- | --- | --- | --- | --- |
| **Title and abstract** | 1 | (*a*) Indicate the study’s design with a commonly used term in the title or the abstract | 1-3 | a retrospective cohort study |
|  |  | (*b*) Provide in the abstract an informative and balanced summary of what was done and what was found | 1-3 | We investigated the influence of impaired renal function on fundus characteristics, pars-plana vitrectomy (PPV) details, and outcomes in patients with proliferative diabetic retinopathy (PDR). In PDR patients, screening for CKD was required before PPV. PDR patients with impaired renal function tended to have more severe ischemic retinal conditions. Comparable PPV outcomes could be obtained in patients with and without impaired renal damage. |
| Introduction | | | |  |
| Background/rationale | 2 | Explain the scientific background and rationale for the investigation being reported | 5 | However, there was little information on the fundus characteristics and operation details in previous work on PDR patients with CKD who underwent PPV.  Herein, we investigated a group of PDR patients who underwent PPV, and we compared the fundus characteristics, operative details, and PPV outcomes between patients with impaired renal function and patients with normal renal function. |
| Objectives | 3 | State specific objectives, including any prespecified hypotheses | 5 | Herein, we investigated a group of PDR patients who underwent PPV, and we compared the fundus characteristics, operative details, and PPV outcomes between patients with impaired renal function and patients with normal renal function. |
| Methods | | | |  |
| Study design | 4 | Present key elements of study design early in the paper | 3,6 | A retrospective cohort study |
| Setting | 5 | Describe the setting, locations, and relevant dates, including periods of recruitment, exposure, follow-up, and data collection | 6 | during 2016.1.1-2017.12.31. |
| Participants | 6 | (*a*) *Cohort study*—Give the eligibility criteria, and the sources and methods of selection of participants. Describe methods of follow-up  *Case-control study*—Give the eligibility criteria, and the sources and methods of case ascertainment and control selection. Give the rationale for the choice of cases and controls  *Cross-sectional study*—Give the eligibility criteria, and the sources and methods of selection of participants | 6 | Inclusion criteria: 1) PDR patients who have been hospitalized for PPV because of a nonclearing VH (persistent or recurrent) or tRD; 2) the high-risk PDR ^14^defined by Early Treatment of Diabetic Retinopathy Study (EDTRS) guidelines were confirmed in operation; 3) available history records and laboratory tests; 4) detailed operation records; 5) detailed follow-up records for at least three months.  The exclusion criteria were as follows: patients with one or more of the following conditions were excluded from the study: 1) vitreous hemorrhage not related to PDR during vitrectomy; 2) eyes with PDR that had PPV for the epiretinal membrane or macular hole; 3) eyes with a history of PPV; 4) a lack of history of DM, or lack of diagnosis information for hypertension (HBP), DM-related cardiocerebral vascular disease or CKD; 5) a lack of operation data for further statistical analysis; 6) a lack of 3-month follow-up; or 7) a history of other renal diseases that caused abnormal renal function. |
|  |  | (*b*) *Cohort study*—For matched studies, give matching criteria and number of exposed and unexposed  *Case-control study*—For matched studies, give matching criteria and the number of controls per case | 6 |  |
| Variables | 7 | Clearly define all outcomes, exposures, predictors, potential confounders, and effect modifiers. Give diagnostic criteria, if applicable | 7-9 | CKD was defined as diabetes with albuminuria, impaired eGFR, or both according to the National Kidney Foundation KDOQI (Kidney Disease Outcomes Quality Initiative) classification^19^. We classified CKD into the following ranges by eGFR: stage 1 (≥ 90 ml/min/1.73 m^2^); stage 2 (60–89 ml/min/1.73 m^2^); stage 3 (30–59 ml/min/1.73 m^2^); stage 4 (15–29 ml/min/1.73 m^2^); and stage 5 (<15 ml/min/1.73 m^2^). Impaired renal function was defined as eGFR<60 ml/min per 1.73 m^2^ (stage 3 or worse than stage 3) ^2^ |
| Data sources/ measurement | 8* | For each variable of interest, give sources of data and details of methods of assessment (measurement). Describe comparability of assessment methods if there is more than one group | *7-9* | the intraoperative bleeding. The severity of intraoperative bleeding was recorded as follows:  grade 0: none;  grade 1: minor bleeding stopping spontaneously or with transient bottle pressure elevation;  grade 2: moderate to severe bleeding requiring endodiathermy or with the formation of broadsheets of clots^15^.  Incomplete scatter photocoagulation was defined as a lack of preexisting photocoagulation in all four quadrants, fewer than 1000 laser spots or the required additional more than 500 laser spots during PPV^16^.  The extension of FVPs was recorded as follows:  grade 0: absence of any adhesion;  grade 1: multiple point adhesions with or without one broad adhesion (broad adhesion was defined as focal adhesion at three sites or more);  grade 2: 1-3 broad adhesions posterior to the equator;  grade 3: 3+ broad adhesions posterior to the equator or two or fewer in quadrants adhesions anterior to the equator;  Grade 4: broad adhesions anterior to the equator in multiple sites^17,18^.  FVPs were classified as predominantly neovascular, mixed neovascular and fibrotic, and predominantly fibrotic^15^. The marked reduction in the caliber of retinal vessels and widespread retinal vessel closure were recorded. |
| Bias | 9 | Describe any efforts to address potential sources of bias | 6,7,9 | Inclusion and exclusion criteria  The t test or Mann–Whitney U test was carried out for continuous variables. The chi-square test or Fisher's exact test was carried out for discrete data |
| Study size | 10 | Explain how the study size was arrived at | Not relevant |  |

Continued on next page

| Quantitative variables | 11 | Explain how quantitative variables were handled in the analyses. If applicable, describe which groupings were chosen and why | 11 | The t test or Mann–Whitney U test was carried out for continuous variables. The chi-square test or Fisher's exact test was carried out for discrete data. |
| --- | --- | --- | --- | --- |
| Statistical methods | 12 | (*a*) Describe all statistical methods, including those used to control for confounding | 11 | Statistical analysis was performed using R version 3.20 (http://www. R-project.org). Patient characteristics were retrieved from their medical charts and recorded in Epidata Entry Client version 2.0.3.15 (http://epidata.dk). |
|  |  | (*b*) Describe any methods used to examine subgroups and interactions | 11 | To investigate the relationship between impaired renal function and PDR characteristics, the patients were divided into two groups: those with impaired renal function and those with normal renal function. The baseline systemic condition, presurgical findings, and ocular findings during PPV were compared between the two groups. Variables with a p value less than 0.3 were further enrolled in a binary backward stepwise logistic regression model. One variable was included or excluded from the model each time by comparing the Akaike information criterion (AIC) value; the model that had the lowest AIC was chosen.  To investigate the influence of CKD on the PPV and PPV outcomes, the variables of PPV management, VA, retinal attachment rate, and the occurrence of POVH and NVG were compared between the two groups. |
|  |  | (*c*) Explain how missing data were addressed | Not relevant |  |
|  |  | (*d*) *Cohort study*—If applicable, explain how loss to follow-up was addressed  *Case-control study*—If applicable, explain how matching of cases and controls was addressed  *Cross-sectional study*—If applicable, describe analytical methods taking account of sampling strategy | 6-7 | Excluded: a lack of 3-month follow-up |
|  |  | (*e*) Describe any sensitivity analyses | Not relevant |  |
| Results | | | | |
| Participants | 13* | (a) Report numbers of individuals at each stage of study—eg numbers potentially eligible, examined for eligibility, confirmed eligible, included in the study, completing follow-up, and analysed | 11 | There were 220 patients enrolled in our study with an average age of 51.8±11.8 years. Among them, 126 (57.3%) were males, and 94 (42.7%) were females. |
|  |  | (b) Give reasons for non-participation at each stage | 12 | There was 5 cases excluded due to failing to follow-up and all of them had stage 1 CKD. |
|  |  | (c) Consider use of a flow diagram | No |  |
| Descriptive data | 14* | (a) Give characteristics of study participants (eg demographic, clinical, social) and information on exposures and potential confounders | 12 | There were 149 patients with stage 1-2 CKD (67.7%) and 71 (32.3%) patients with impaired renal function and stage 3-5 CKD |
|  |  | (b) Indicate number of participants with missing data for each variable of interest | Not relevant |  |
|  |  | (c) *Cohort study*—Summarise follow-up time (eg, average and total amount) | 16 | During a median follow-up of 29 weeks, ranging from 24 to 40 weeks |
| Outcome data | 15* | *Cohort study*—Report numbers of outcome events or summary measures over time | *16-17* | 46.4% of patients with impaired renal function had VA increased for more than two lines at their 3-month follow-up, similar to the 54.3% of patients with normal renal function (p=0.34); 42.2% of patients with impaired renal function had stable (VA changes were less than two lines) 3-month follow-up VA, similar to the 56.4% patients with normal renal function (p=0.07).  In 74 patients with silicone oil tamponade, macular reattachment was found in all patients at silicone oil removal. Four patients with impaired renal function and three patients with normal renal function had persistent macular edema. In 220 patients, two patients with impaired renal function and one with normal renal function had secondary PPV for recurrent retinal detachment; the retinal attachment rates were 97.2% and 99.3%, respectively.  Four patients with impaired renal function and eight with normal renal function had POVH (p=0.99). POVH occurred after restarting antiplatelet therapy.  No patient in our study developed NVG during the follow-up period. |
|  |  | *Case-control study—*Report numbers in each exposure category, or summary measures of exposure |  |  |
|  |  | *Cross-sectional study—*Report numbers of outcome events or summary measures |  |  |
| Main results | 16 | (*a*) Give unadjusted estimates and, if applicable, confounder-adjusted estimates and their precision (eg, 95% confidence interval). Make clear which confounders were adjusted for and why they were included | 15 | Logistic regression showed that factors related to impaired renal function were the presence of HTN (3.40[1.58-7.29], p=0.002), preoperative incomplete PRP (3.18[1.50-6.72], p=0.002), presence of grade 3 FVP (8.19[3.43-19.54], p<0.001), and presence of extensive retinal vascular closure (3.40[1.54-7.52], p=0.002) (AIC=203.4, AUC=0.854). |
|  |  | (*b*) Report category boundaries when continuous variables were categorized | 14 | Table 1. The baseline and intraoperative characteristics of PDR patients by the presence of impaired renal function |
|  |  | (*c*) If relevant, consider translating estimates of relative risk into absolute risk for a meaningful time period | Not relevant |  |

Continued on next page

| Other analyses | 17 | Report other analyses done—eg analyses of subgroups and interactions, and sensitivity analyses | 15 | Logistic regression showed that factors related to impaired renal function were the presence of HTN (3.40[1.58-7.29], p=0.002), preoperative incomplete PRP (3.18[1.50-6.72], p=0.002), presence of grade 3 FVP (8.19[3.43-19.54], p<0.001), and presence of extensive retinal vascular closure (3.40[1.54-7.52], p=0.002) (AIC=203.4, AUC=0.854) |
| --- | --- | --- | --- | --- |
| Discussion | | | | |
| Key results | 18 | Summarise key results with reference to study objectives | 17 | Previous investigations on the relationship between DR and CKD were carried out in DM patients with transparent optic media. These studies often excluded PDR patients with VH due to failure to obtain a clear fundus photograph. We enrolled PDR patients with VH who underwent PPV and investigated the fundus characteristics of PDR patients after removing the VH by PPV. We found that PDR patients with impaired renal function had specific FVP and retinal vascular characteristics. Correspondingly, specific operative details were different between the two groups. Finally, after proper operative management, we found that PDR patients with impaired renal function could achieve comparable operative outcomes compared with PDR patients with normal renal function. |
| Limitations | 19 | Discuss limitations of the study, taking into account sources of potential bias or imprecision. Discuss both direction and magnitude of any potential bias | 22-23 | Previous research showed that 44-72% of VH in PDR patients could resolve after IV-anti-VEGF agents in a follow-up of 16-52 weeks; only 7.04-33.0% of patients required PPV^40-43^. Since our study was a retrospective study with a relatively low percentage of patients who received IV-anti-VEGF agent after VH occurrence (6.5%, 7.5%), we could not show the influence of anti-VEFG agent injection on the resolution of VH and the chances of gaining a second PRP. The high prevalence of severe FVP in patients with impaired renal function in our patients suggested that the observation for VH absorption after IV-anti-VEGF agents in PDR patients combined with impaired renal function should not be too long in case of progression of tRD. The safe length of the observation period in patients with CKD after IV anti-VEGF agents should be further investigated.  We only had serum creatinine, blood urine nitrogen, and urine protein tests; we did not have a urinary albumin‐to‐creatinine ratio test. We could not show the influence of CKD with relatively normal renal function on the PDR characteristics or PPV details.  Incomplete PRP is related to the progression of PDR^44,45^ and the occurrence of VH^46^, POVH^27,^ and postoperative NVI ^31-33^. We reported a low prevalence of completion of PRP (3/71, 3/149) before PPV and a high number of laser points during the surgery (980, 1092). Nevertheless, due to the retrospective characteristics, we could not show the reason for incomplete PRP in our patients. The reasons for the incomplete PRP may be unawareness of DR or developing VH in high-risk PDR. Further investigation of the reason for incomplete PRP may provide more information on the influence of CKD on DR patients' treatment before ophthalmic surgery. |
| Interpretation | 20 | Give a cautious overall interpretation of results considering objectives, limitations, multiplicity of analyses, results from similar studies, and other relevant evidence | 17-23 | our results suggested that ophthalmologists should pay extra attention to the screening and treatment of CKD when dealing with fundus abnormalities in PDR patients who underwent PPV. We extended the relationship of CKD and DR to patients who underwent PPV with unclear optic media, and our results showed that impaired renal function was related to more severe ischemic retinal changes, including retinal vessel closure and board FVP. It suggests that more aggressive treatment for CKD in patients with DR should be applied, and more attention should be given to DR progression in CKD patients. Our data suggested that subretinal fluid drainage in PPV in PDR patients, especially in patients with impaired renal function, should be performed considering the risk of NVG in addition to visual outcomes. Our results suggested that preoperative IV-anti-VEGF agents and the use of endodiathermy in cases with extensive FVP may be practical to lessen the chance of POVH. |
| Generalisability | 21 | Discuss the generalisability (external validity) of the study results | 22-23 | We showed that the prevalence of impaired renal function in PDR patients who underwent PPV was high, the awareness of the renal condition among PDR patients was low, and screening for CKD was required before PPV. We also found that PDR patients with impaired renal function had a high prevalence of extensive FVP, macular-involved tRD, and retinal vessel closure. In patients with extensive tRD, subretinal fluid drainage and photocoagulation are recommended to lessen the chance of NVG; moreover, pre-PPV anti-VEGF agents and endodiathermy in PPV could lessen the chance of POVH. In addition, patients with impaired renal function can achieve VA results and PPV outcomes comparable to those of patients with normal renal function after proper management during PPV. |
| Other information | |  | | |
| Funding | 22 | Give the source of funding and the role of the funders for the present study and, if applicable, for the original study on which the present article is based | 2 | The work was supported by The Fund for Beijing Science & Technology Development of TCM (No.JJ-2020-25) |

*Give information separately for cases and controls in case-control studies and, if applicable, for exposed and unexposed groups in cohort and cross-sectional studies.

**Note:** An Explanation and Elaboration article discusses each checklist item and gives methodological background and published examples of transparent reporting. The STROBE checklist is best used in conjunction with this article (freely available on the Web sites of PLoS Medicine at http://www.plosmedicine.org/, Annals of Internal Medicine at http://www.annals.org/, and Epidemiology at http://www.epidem.com/). Information on the STROBE Initiative is available at www.strobe-statement.org.
