# Supplementary material for: Ocular findings, surgery details and outcomes in proliferative diabetic retinopathy patients with chronic kidney disease
Source: PLoS One. 2022 Oct 21;17(10):e0273133. doi: 10.1371/journal.pone.0273133 (PMC9586353; doi:10.1371/journal.pone.0273133)
Supplement: Attachment — Submitted filename: answer for reviewers and editor.docx [file pone.0273133.s002.docx]

Dear editors and reviewers:

Thank you for your valuable suggestion. We have made changes according to your suggestions and marked them with red color.

journal Requirements:

When submitting your revision, we need you to address these additional requirements.

1. Please ensure that your manuscript meets PLOS ONE's style requirements, including those for file naming. The PLOS ONE style templates can be found at https://journals.plos.org/plosone/s/file?id=wjVg/PLOSOne_formatting_sample_main_body.pdf and <https://journals.plos.org/plosone/s/file?id=ba62/PLOSOne_formatting_sample_title_authors_affiliations.pdf>.

**We have modified our manuscript to meet PLOS ONE's style.**

2. We note that the grant information you provided in the 'Funding Information' and 'Financial Disclosure' sections do not match. When you resubmit, please ensure that you provide the correct grant numbers for the awards you received for your study in the 'Funding Information' section.

**We provided the correct grant number for the awards(line16).**

3. We note that you have stated that you will provide repository information for your data at acceptance. Should your manuscript be accepted for publication, we will hold it until you provide the relevant accession numbers or DOIs necessary to access your data. If you wish to make changes to your Data Availability statement, please describe these changes in your cover letter and we will update your Data Availability statement to reflect the information you provide.

**The DOIs have been included in the manuscript (line 617).**

4. Please include your full ethics statement in the 'Methods’ section of your manuscript file. In your statement, please include the full name of the IRB or ethics committee who approved or waived your study, as well as whether or not you obtained informed written or verbal consent. If consent was waived for your study, please include this information in your statement as well.

**We had included the ethics issue in the Methods section (line76-81).**

Additional Editor Comments:

I thought this paper was a very meaningful report from a clinical perspective for ophthalmologists.
I think the points made by the reviewers are very much on point.
I hope you accept the reviewers' points and that this paper will improve.

Thank you.

Major comments:

1. Have you accessed histories of DM control and compliance toward DMR treatments before operations by HbA1c, frequency of outpatient appointment, amount of leftover medicine, and so on?

**We did not involve the control of DM and compliance information in our study. The compliance was poor, as we mentioned in the manuscript that 85.4% of patients did not have CKD screening before the preoperative assessment(line 230). We added the HbA1C result in result (line225).**

**We did not have the frequency of outpatient appointments and amount of leftover medicine due to the retrospective character of our study. We added it in the discussion section(line 335-339).**

Ischemic retinal changes may be related to CKD, but bad control of DM and patients’ non-cooperative behavior can be confounder both to the progression of DMR to PDR and deterioration of renal function. In addition to this, severely impaired renal function-- stage 4 and 5, for example-- also can affect patients’ commuting to your eye hospital because of hemodialysis schedules and poor physical condition. It may be difficult to quantify the patients’ compliance and cooperation, but I would appreciate it if you took them into consideration in some way.
Related question: Were the incomplete PRPs intentional by eye doctor or because of patients’ low attendance to your eye hospital?

**Thank you for your suggestion. We investigated the influence of poor compliance of patients on the severity of DR in both groups of patients and found that poor compliance indicated by low screening CKD rate and high HbA1c was presented in both groups (Table1). We added the number of patients who did not have the chance to complete PRP at their first presentation due to VH (Table1), and the result suggested the rate of proper PRP in time was low. We also added we could not tell the reason for incomplete PRP in patients who developed VH after initiation of PRP in the discussion section (line446-451).**

2. How many patients took antiplatelet and / or anticoagulant medicine and how long time before operation did you stopped it before operation? Table1 showed among patients with impaired renal function the number of combined stroke was larger than and the number of combined coronary heart disease was as large as the numbers among patients with normal renal function. Their medications could have affected intraoperative bleedings.

**We added the information on the long-term use of antiplatelet or anticoagulant medicine for long-term coexisted DM complications, such as stroke, coronary heart disease, and CKD, and adjustments of those medicine during PPV in the table and result section (line117-130, Table1.
The percentage of patients with long-term use of anticoagulation or antiplatelet medication was low. Most the patients adjusted the anticoagulant or antiplatelet medication before PPV. We could not show the role of antiplatelet or anticoagulation medicine on intraoperative bleeding (line 268-274). We added it in the discussion section(line 419-421).**
Minor comments:
Table1: presur”G”ery ocular findings and ocular medical history may be correct.
**Changes have been made in Table 1.**

Reviewer #2: Thank you for allowing me to review your paper.
It is a very good paper with a clinical point of view, and it would be very meaningful to publish it. However, at the moment it seems that some work is needed, mainly on statistical issues.

#1 First, please describe in the inclusion criteria whether dialysis patients are included among renal disorders.

**Changes have been made to the inclusion criteria. The dialysis patients were included (line 89).**

#2 Next, if the study period is 3 months, change in visual acuity at 3 months.
It should be graphed and compared to before treatment. In addition, it is necessary to describe the presence or absence of anatomical reversion of the macula in the group in which OCT imaging was possible. The rate of onset of neovascular glaucoma and PVR, which are the most feared complications, would also be good indicators.

**Fig2 and Table3 have been added. The preoperative presence of dense VH, tractional macular retinal detachment, and macular involved FVP may obscure the OCT image. We failed to obtain the preoperative OCT image in parts of our patients. The absence of anatomical reversion of macular by OCT assessment was explained in the discussion section (line454-459). The rate of NVG and recurrent RD had been added in the result section (line300-302)**

#3 In addition, multivariate analysis of final visual acuity and anatomical restoration of the macula, CKD grade, age, gender, presence or absence of PRP, presence or absence of IVR, visual acuity at first visit, etc. is required. This will ensure the success of this paper.
**We compared the final VA as the number of patients with VA increased, VA was stable between patients with impaired renal function and patients with normal renal function. There was no significant difference (line290-293). We did not have the anatomical restoration of macular (we added in limitation(line 454-459) )**

**According to your suggestion, we performed the comparison between patients with increased final VA and patients without. However, we did not find the relationship between the final VA and the CKD grade, age, gender, PRP, IVR, preoperative VA by multivariate analysis and added it in the result section and discussed the final VA in the discussion section.**

. **(line 285-292, Table3, Fig2, line 383-383).**

Thank you for your consideration. Hoping to hear from you soon.

Best regards,

Meng ZHAO

Beijing Tongren Eye center
