# Supplementary material for: Ocular findings, surgery details and outcomes in proliferative diabetic retinopathy patients with chronic kidney disease
Source: PLoS One. 2022 Oct 21;17(10):e0273133. doi: 10.1371/journal.pone.0273133 (PMC9586353; doi:10.1371/journal.pone.0273133)
Supplement: Peer review history

# Peer review: Decision Letter 0

**DOI:** 10.1371/journal.pone.0273133.r001

**Authors:** Daisuke Nagasato

29 Aug 2022

PONE-D-22-21450Ocular findings, surgery details and outcomes in proliferative diabetic retinopathy patients with chronic kidney diseasePLOS ONE

Dear Dr. ZHAO,

Thank you for submitting your manuscript to PLOS ONE. After careful consideration, we feel that it has merit but does not fully meet PLOS ONE’s publication criteria as it currently stands. Therefore, we invite you to submit a revised version of the manuscript that addresses the points raised during the review process.

Please submit your revised manuscript by Oct 12 2022 11:59PM. If you will need more time than this to complete your revisions, please reply to this message or contact the journal office at plosone@plos.org. When you're ready to submit your revision, log on to https://www.editorialmanager.com/pone/ and select the 'Submissions Needing Revision' folder to locate your manuscript file.

Please include the following items when submitting your revised manuscript:

- A rebuttal letter that responds to each point raised by the academic editor and reviewer(s). You should upload this letter as a separate file labeled 'Response to Reviewers'.
- A marked-up copy of your manuscript that highlights changes made to the original version. You should upload this as a separate file labeled 'Revised Manuscript with Track Changes'.
- An unmarked version of your revised paper without tracked changes. You should upload this as a separate file labeled 'Manuscript'.

If you would like to make changes to your financial disclosure, please include your updated statement in your cover letter. Guidelines for resubmitting your figure files are available below the reviewer comments at the end of this letter.

If applicable, we recommend that you deposit your laboratory protocols in protocols.io to enhance the reproducibility of your results. Protocols.io assigns your protocol its own identifier (DOI) so that it can be cited independently in the future. For instructions see: https://journals.plos.org/plosone/s/submission-guidelines#loc-laboratory-protocols. Additionally, PLOS ONE offers an option for publishing peer-reviewed Lab Protocol articles, which describe protocols hosted on protocols.io. Read more information on sharing protocols at https://plos.org/protocols?utm_medium=editorial-email&utm_source=authorletters&utm_campaign=protocols.

We look forward to receiving your revised manuscript.

Kind regards,

Daisuke Nagasato

Academic Editor

PLOS ONE

Journal Requirements:

When submitting your revision, we need you to address these additional requirements.

1. Please ensure that your manuscript meets PLOS ONE's style requirements, including those for file naming. The PLOS ONE style templates can be found at 

https://journals.plos.org/plosone/s/file?id=wjVg/PLOSOne_formatting_sample_main_body.pdf and 

https://journals.plos.org/plosone/s/file?id=ba62/PLOSOne_formatting_sample_title_authors_affiliations.pdf.

2. We note that the grant information you provided in the ‘Funding Information’ and ‘Financial Disclosure’ sections do not match. 

When you resubmit, please ensure that you provide the correct grant numbers for the awards you received for your study in the ‘Funding Information’ section.

3. We note that you have stated that you will provide repository information for your data at acceptance. Should your manuscript be accepted for publication, we will hold it until you provide the relevant accession numbers or DOIs necessary to access your data. If you wish to make changes to your Data Availability statement, please describe these changes in your cover letter and we will update your Data Availability statement to reflect the information you provide.

4. Please include your full ethics statement in the ‘Methods’ section of your manuscript file. In your statement, please include the full name of the IRB or ethics committee who approved or waived your study, as well as whether or not you obtained informed written or verbal consent. If consent was waived for your study, please include this information in your statement as well. 

Additional Editor Comments:

I thought this paper was a very meaningful report from a clinical perspective for ophthalmologists.

I think the points made by the reviewers are very much on point.

I hope you accept the reviewers' points and that this paper will improve.

Thank you.

[Note: HTML markup is below. Please do not edit.]

Reviewers' comments:

Reviewer's Responses to Questions

**Comments to the Author**

1. Is the manuscript technically sound, and do the data support the conclusions?

The manuscript must describe a technically sound piece of scientific research with data that supports the conclusions. Experiments must have been conducted rigorously, with appropriate controls, replication, and sample sizes. The conclusions must be drawn appropriately based on the data presented. 

Reviewer #1: Yes

Reviewer #2: Yes

**********

2. Has the statistical analysis been performed appropriately and rigorously? 

Reviewer #1: Yes

Reviewer #2: No

**********

3. Have the authors made all data underlying the findings in their manuscript fully available?

The PLOS Data policy requires authors to make all data underlying the findings described in their manuscript fully available without restriction, with rare exception (please refer to the Data Availability Statement in the manuscript PDF file). The data should be provided as part of the manuscript or its supporting information, or deposited to a public repository. For example, in addition to summary statistics, the data points behind means, medians and variance measures should be available. If there are restrictions on publicly sharing data—e.g. participant privacy or use of data from a third party—those must be specified.

Reviewer #1: Yes

Reviewer #2: Yes

**********

4. Is the manuscript presented in an intelligible fashion and written in standard English?

PLOS ONE does not copyedit accepted manuscripts, so the language in submitted articles must be clear, correct, and unambiguous. Any typographical or grammatical errors should be corrected at revision, so please note any specific errors here.

Reviewer #1: Yes

Reviewer #2: Yes

**********

5. Review Comments to the Author

Please use the space provided to explain your answers to the questions above. You may also include additional comments for the author, including concerns about dual publication, research ethics, or publication ethics. (Please upload your review as an attachment if it exceeds 20,000 characters)

Reviewer #1: Dear authors,

This paper compares those having low renal function with those having normal renal function among proliferative diabetic retinopathy (PDR) patients having gone through pars-plana vitrectomy (PPV) by retrospective cohort design. Subjects of comparison are systemic and ocular findings by pre-operation screening, findings during operations and during follow-ups. This investigation found that PDR eyes of those having low renal function tend to show more severe retinal ischemic findings and occur intraoperative bleedings.

The results obtained emphasizes the importance of grasping the renal function of patients with diabetic retinopathy and will be educational for ophthalmologists taking part in PPV to PDR eyes.

I recommend that this paper be accepted after minor revision.

Major comments:

1. Have you accessed histories of DM control and compliance toward DMR treatments before operations by HbA1c, frequency of outpatient appointment, amount of leftover medicine, and so on?

Ischemic retinal changes may be related to CKD, but bad control of DM and patients’ non-cooperative behavior can be confounder both to the progression of DMR to PDR and deterioration of renal function. In addition to this, severely impaired renal function-- stage 4 and 5, for example-- also can affect patients’ commuting to your eye hospital because of hemodialysis schedules and poor physical condition. It may be difficult to quantify the patients’ compliance and cooperation, but I would appreciate it if you took them into consideration in some way.

Related question: Were the incomplete PRPs intentional by eye doctor or because of patients’ low attendance to your eye hospital?

2. How many patients took anti-platelet and / or anti-coagulant medicine and how long time before operation did you stopped it before operation? Table1 showed among patients with impaired renal function the number of combined stroke was larger than and the number of combined coronary heart disease was as large as the numbers among patients with normal renal function. Their medications could have affected intraoperative bleedings.

Minor comments:

Table1: presur”G”ery ocular findings and ocular medical history may be correct.

Sincerely,

Reviewer #2: Thank you for allowing me to review your paper.

It is a very good paper with a clinical point of view, and it would be very meaningful to publish it. However, at the moment it seems that some work is needed, mainly on statistical issues.

#1 First, please describe in the inclusion criteria whether dialysis patients are included among renal disorders.

#2 Next, if the study period is 3 months, change in visual acuity at 3 months.

It should be graphed and compared to before treatment. In addition, it is necessary to describe the presence or absence of anatomical reversion of the macula in the group in which OCT imaging was possible. The rate of onset of neovascular glaucoma and PVR, which are the most feared complications, would also be good indicators.

#3 In addition, multivariate analysis of final visual acuity and anatomical restoration of the macula, CKD grade, age, gender, presence or absence of PRP, presence or absence of IVR, visual acuity at first visit, etc. is required. This will ensure the success of this paper.

Let's add a discussion based on them.

**********

6. PLOS authors have the option to publish the peer review history of their article (what does this mean?). If published, this will include your full peer review and any attached files.

If you choose “no”, your identity will remain anonymous but your review may still be made public.

**Do you want your identity to be public for this peer review?** For information about this choice, including consent withdrawal, please see our Privacy Policy.

Reviewer #1: **Yes: **Hayato Tanaka

Reviewer #2: **Yes: **Mizuki　Tagami

**********

[NOTE: If reviewer comments were submitted as an attachment file, they will be attached to this email and accessible via the submission site. Please log into your account, locate the manuscript record, and check for the action link "View Attachments". If this link does not appear, there are no attachment files.]

While revising your submission, please upload your figure files to the Preflight Analysis and Conversion Engine (PACE) digital diagnostic tool, https://pacev2.apexcovantage.com/. PACE helps ensure that figures meet PLOS requirements. To use PACE, you must first register as a user. Registration is free. Then, login and navigate to the UPLOAD tab, where you will find detailed instructions on how to use the tool. If you encounter any issues or have any questions when using PACE, please email PLOS at figures@plos.org. Please note that Supporting Information files do not need this step.

# Author comment: Author response to Decision Letter 0

**DOI:** 10.1371/journal.pone.0273133.r002

30 Sep 2022

Dear editors and reviewers:

Thank you for your valuable suggestion. We have made changes according to your suggestions and marked them with red color. 

journal Requirements:

When submitting your revision, we need you to address these additional requirements.

1. Please ensure that your manuscript meets PLOS ONE's style requirements, including those for file naming. The PLOS ONE style templates can be found at https://journals.plos.org/plosone/s/file?id=wjVg/PLOSOne_formatting_sample_main_body.pdf and https://journals.plos.org/plosone/s/file?id=ba62/PLOSOne_formatting_sample_title_authors_affiliations.pdf.

We have modified our manuscript to meet PLOS ONE's style.

2. We note that the grant information you provided in the 'Funding Information' and 'Financial Disclosure' sections do not match. When you resubmit, please ensure that you provide the correct grant numbers for the awards you received for your study in the 'Funding Information' section.

We provided the correct grant number for the awards(line16).

3. We note that you have stated that you will provide repository information for your data at acceptance. Should your manuscript be accepted for publication, we will hold it until you provide the relevant accession numbers or DOIs necessary to access your data. If you wish to make changes to your Data Availability statement, please describe these changes in your cover letter and we will update your Data Availability statement to reflect the information you provide.

The DOIs have been included in the manuscript (line 617).

4. Please include your full ethics statement in the 'Methods’ section of your manuscript file. In your statement, please include the full name of the IRB or ethics committee who approved or waived your study, as well as whether or not you obtained informed written or verbal consent. If consent was waived for your study, please include this information in your statement as well. 

We had included the ethics issue in the Methods section (line76-81).

Additional Editor Comments:

I thought this paper was a very meaningful report from a clinical perspective for ophthalmologists.

I think the points made by the reviewers are very much on point.

I hope you accept the reviewers' points and that this paper will improve.

Thank you.

Major comments:

1. Have you accessed histories of DM control and compliance toward DMR treatments before operations by HbA1c, frequency of outpatient appointment, amount of leftover medicine, and so on?

We did not involve the control of DM and compliance information in our study. The compliance was poor, as we mentioned in the manuscript that 85.4% of patients did not have CKD screening before the preoperative assessment(line 230). We added the HbA1C result in result (line225).

We did not have the frequency of outpatient appointments and amount of leftover medicine due to the retrospective character of our study. We added it in the discussion section(line 335-339).

Ischemic retinal changes may be related to CKD, but bad control of DM and patients’ non-cooperative behavior can be confounder both to the progression of DMR to PDR and deterioration of renal function. In addition to this, severely impaired renal function-- stage 4 and 5, for example-- also can affect patients’ commuting to your eye hospital because of hemodialysis schedules and poor physical condition. It may be difficult to quantify the patients’ compliance and cooperation, but I would appreciate it if you took them into consideration in some way.

Related question: Were the incomplete PRPs intentional by eye doctor or because of patients’ low attendance to your eye hospital?

Thank you for your suggestion. We investigated the influence of poor compliance of patients on the severity of DR in both groups of patients and found that poor compliance indicated by low screening CKD rate and high HbA1c was presented in both groups (Table1). We added the number of patients who did not have the chance to complete PRP at their first presentation due to VH (Table1), and the result suggested the rate of proper PRP in time was low. We also added we could not tell the reason for incomplete PRP in patients who developed VH after initiation of PRP in the discussion section (line446-451).

2. How many patients took antiplatelet and / or anticoagulant medicine and how long time before operation did you stopped it before operation? Table1 showed among patients with impaired renal function the number of combined stroke was larger than and the number of combined coronary heart disease was as large as the numbers among patients with normal renal function. Their medications could have affected intraoperative bleedings.

We added the information on the long-term use of antiplatelet or anticoagulant medicine for long-term coexisted DM complications, such as stroke, coronary heart disease, and CKD, and adjustments of those medicine during PPV in the table and result section (line117-130, Table1. 

The percentage of patients with long-term use of anticoagulation or antiplatelet medication was low. Most the patients adjusted the anticoagulant or antiplatelet medication before PPV. We could not show the role of antiplatelet or anticoagulation medicine on intraoperative bleeding (line 268-274). We added it in the discussion section(line 419-421). 

Minor comments:

Table1: presur”G”ery ocular findings and ocular medical history may be correct.

Changes have been made in Table 1.

Reviewer #2: Thank you for allowing me to review your paper.

It is a very good paper with a clinical point of view, and it would be very meaningful to publish it. However, at the moment it seems that some work is needed, mainly on statistical issues.

#1 First, please describe in the inclusion criteria whether dialysis patients are included among renal disorders.

Changes have been made to the inclusion criteria. The dialysis patients were included (line 89). 

#2 Next, if the study period is 3 months, change in visual acuity at 3 months.

It should be graphed and compared to before treatment. In addition, it is necessary to describe the presence or absence of anatomical reversion of the macula in the group in which OCT imaging was possible. The rate of onset of neovascular glaucoma and PVR, which are the most feared complications, would also be good indicators.

Fig2 and Table3 have been added. The preoperative presence of dense VH, tractional macular retinal detachment, and macular involved FVP may obscure the OCT image. We failed to obtain the preoperative OCT image in parts of our patients. The absence of anatomical reversion of macular by OCT assessment was explained in the discussion section (line454-459). The rate of NVG and recurrent RD had been added in the result section (line300-302)

#3 In addition, multivariate analysis of final visual acuity and anatomical restoration of the macula, CKD grade, age, gender, presence or absence of PRP, presence or absence of IVR, visual acuity at first visit, etc. is required. This will ensure the success of this paper.

We compared the final VA as the number of patients with VA increased, VA was stable between patients with impaired renal function and patients with normal renal function. There was no significant difference (line290-293). We did not have the anatomical restoration of macular (we added in limitation(line 454-459) )

According to your suggestion, we performed the comparison between patients with increased final VA and patients without. However, we did not find the relationship between the final VA and the CKD grade, age, gender, PRP, IVR, preoperative VA by multivariate analysis and added it in the result section and discussed the final VA in the discussion section.

. (line 285-292, Table3, Fig2, line 383-383).

________________________________________

Thank you for your consideration. Hoping to hear from you soon.

Best regards,

Meng ZHAO

 Beijing Tongren Eye center

Attachment Submitted filename: answer for reviewers and editor.docx Click here for additional data file.

# Peer review: Decision Letter 1

**DOI:** 10.1371/journal.pone.0273133.r003

**Authors:** Daisuke Nagasato

10 Oct 2022

Ocular findings, surgery details and outcomes in proliferative diabetic retinopathy patients with chronic kidney disease

PONE-D-22-21450R1

Dear Dr. ZHAO,

We’re pleased to inform you that your manuscript has been judged scientifically suitable for publication and will be formally accepted for publication once it meets all outstanding technical requirements.

Within one week, you’ll receive an e-mail detailing the required amendments. When these have been addressed, you’ll receive a formal acceptance letter and your manuscript will be scheduled for publication.

An invoice for payment will follow shortly after the formal acceptance. To ensure an efficient process, please log into Editorial Manager at http://www.editorialmanager.com/pone/, click the 'Update My Information' link at the top of the page, and double check that your user information is up-to-date. If you have any billing related questions, please contact our Author Billing department directly at authorbilling@plos.org.

If your institution or institutions have a press office, please notify them about your upcoming paper to help maximize its impact. If they’ll be preparing press materials, please inform our press team as soon as possible -- no later than 48 hours after receiving the formal acceptance. Your manuscript will remain under strict press embargo until 2 pm Eastern Time on the date of publication. For more information, please contact onepress@plos.org.

Kind regards,

Daisuke Nagasato

Academic Editor

PLOS ONE

Additional Editor Comments (optional):

Reviewers' comments:

Reviewer's Responses to Questions

**Comments to the Author**

1. If the authors have adequately addressed your comments raised in a previous round of review and you feel that this manuscript is now acceptable for publication, you may indicate that here to bypass the “Comments to the Author” section, enter your conflict of interest statement in the “Confidential to Editor” section, and submit your "Accept" recommendation.

Reviewer #1: All comments have been addressed

Reviewer #2: (No Response)

**********

2. Is the manuscript technically sound, and do the data support the conclusions?

The manuscript must describe a technically sound piece of scientific research with data that supports the conclusions. Experiments must have been conducted rigorously, with appropriate controls, replication, and sample sizes. The conclusions must be drawn appropriately based on the data presented. 

Reviewer #1: Yes

Reviewer #2: Yes

**********

3. Has the statistical analysis been performed appropriately and rigorously? 

Reviewer #1: Yes

Reviewer #2: Yes

**********

4. Have the authors made all data underlying the findings in their manuscript fully available?

The PLOS Data policy requires authors to make all data underlying the findings described in their manuscript fully available without restriction, with rare exception (please refer to the Data Availability Statement in the manuscript PDF file). The data should be provided as part of the manuscript or its supporting information, or deposited to a public repository. For example, in addition to summary statistics, the data points behind means, medians and variance measures should be available. If there are restrictions on publicly sharing data—e.g. participant privacy or use of data from a third party—those must be specified.

Reviewer #1: Yes

Reviewer #2: Yes

**********

5. Is the manuscript presented in an intelligible fashion and written in standard English?

PLOS ONE does not copyedit accepted manuscripts, so the language in submitted articles must be clear, correct, and unambiguous. Any typographical or grammatical errors should be corrected at revision, so please note any specific errors here.

Reviewer #1: Yes

Reviewer #2: Yes

**********

6. Review Comments to the Author

Please use the space provided to explain your answers to the questions above. You may also include additional comments for the author, including concerns about dual publication, research ethics, or publication ethics. (Please upload your review as an attachment if it exceeds 20,000 characters)

Reviewer #1: I appreciated your response telling us the information about Diabetes Mellitus history investigation, affection of anti-platelet. These information helped our understanding of your research.

Reviewer #2: I think that it is well corrected and suitable for publication.　The reviewer feel that there is a strong relationship between renal function and intraoperative bleeding and postoperative ocular ischemic events.

Thank you for your good consideration.

**********

7. PLOS authors have the option to publish the peer review history of their article (what does this mean?). If published, this will include your full peer review and any attached files.

If you choose “no”, your identity will remain anonymous but your review may still be made public.

**Do you want your identity to be public for this peer review?** For information about this choice, including consent withdrawal, please see our Privacy Policy.

Reviewer #1: No

Reviewer #2: **Yes: **MIzuki Tagami

**********

# Editor report: Acceptance letter

**DOI:** 10.1371/journal.pone.0273133.r004

**Authors:** Daisuke Nagasato

13 Oct 2022

PONE-D-22-21450R1 

Ocular findings, surgery details and outcomes in proliferative diabetic retinopathy patients with chronic kidney disease 

Dear Dr. Zhao:

I'm pleased to inform you that your manuscript has been deemed suitable for publication in PLOS ONE. Congratulations! Your manuscript is now with our production department. 

If your institution or institutions have a press office, please let them know about your upcoming paper now to help maximize its impact. If they'll be preparing press materials, please inform our press team within the next 48 hours. Your manuscript will remain under strict press embargo until 2 pm Eastern Time on the date of publication. For more information please contact onepress@plos.org.

If we can help with anything else, please email us at plosone@plos.org. 

Thank you for submitting your work to PLOS ONE and supporting open access. 

Kind regards, 

PLOS ONE Editorial Office Staff

on behalf of

Dr. Daisuke Nagasato 

Academic Editor

PLOS ONE
